# Evaluation of finger millet (*Eleusine coracana* (L.) Gaertn.) in multi-environment trials using enhanced statistical models

**DOI:** 10.1371/journal.pone.0277499

**Published:** 2023-02-01

**Authors:** Kassahun Tesfaye, Tesfaye Alemu, Tarekegn Argaw, Santie de Villiers, Ermias Assefa

**Affiliations:** 1 Department of Microbial, Cellular and Molecular Biology, Addis Ababa University, Addis Ababa, Ethiopia; 2 Bio and Emerging Technology Institute, Addis Ababa, Ethiopia; 3 Department of Biometrics, Ethiopian Institute of Agricultural Research (EIAR), Addis Ababa, Ethiopia; 4 Department of Biochemistry and Biotechnology and Pwani University Biosciences Research Centre, Pwani University, Kilifi, Kenya; Nepal Agricultural Research Council, NEPAL

## Abstract

Spatial variation and genotype by environment (GxE) interaction are common in varietal selection field trials and pose a significant challenge for plant breeders when comparing the genetic potential of different varieties. Efficient statistical methods must be employed for the evaluation of finger millet breeding trials to accurately select superior varieties that contribute to agricultural productivity. The objective of this study was to improve selection strategies in finger millet breeding in Ethiopia through modeling of spatial field trends and the GxE interaction. A dataset of seven multi-environment trials (MET) conducted in randomized complete block design (RCBD) with two replications laid out in rectangle (row x column) arrays of plots was used in this study. The results revealed that, under the linear mixed model, the spatial and factor analytic (FA) models were efficient methods of data analysis for this study, and this was demonstrated with evidence of heritability measure. We found two clusters of correlated environments that helped to select superior and stable varieties through ranking average Best Linear Unbiased Predictors (BLUPs) within clusters. The first cluster was chosen because it contained a greater number of environments with high heritability. Based on this cluster, Bako-09, 203439, 203325, and 203347 were the top four varieties with relatively high yield performance and stability across correlated environments. Hence, scaling up the use of this efficient analysis method will improve the selection of superior finger millet varieties.

## Introduction

Cereals are the main source of nutrition for the majority of people on earth. Millets are small-seeded species of cereal crops that are members of the Gramineae family [1]. Small-seeded grasses produced for food, feed, or forage comprise the diverse group of millets of which finger millet (*Eleusine coracana* (L.) Gaertn.) is the fourth most important after sorghum, pearl millet, and foxtail millet [2, 3]. Finger millet is an allotetraploid (2n = 4X = 36, AABB) belonging to the family Poaceae and genus Eleusine [4].

Finger millet is the most climate resilient millet and can be grown under a wide spectrum of extreme climatic conditions. Thus, they can be termed “farmer friendly”, providing better returns in comparison to other crops when subjected to changing climatic conditions [5]. Globally, 3,834,021 tons of finger millet grain are produced per annum [6]. In Ethiopia, 480,852 hectares of finger millet yield 1,327,267 tons annually [7].

Finger millet stands unique among the cereals such as barley, rye and oats with higher nutritional contents and has outstanding properties as a subsistence food crop. It is rich in calcium (0.34%), dietary fiber (18%), phytates (0.48%), protein (6%–13%) minerals (2.5%–3.5%), and phenolics (0.3%–3%) [8]. In Ethiopia, finger millet is used to make porridge, pancakes, injera (thin flatten fermented bread), and traditional alcoholic beverages [9].

Even though finger millet is crucial for livelihoods and food security, its productivity is far below the potential yield attained under experimental settings [10]. This is attributed to a variety of biotic and abiotic challenges coupled with socioeconomic limitations to adopt modern farming technologies. Finger millet blast disease, the most damaging constraint, is caused by Pyricularia grisea (Magnaporthe grisea), and can reduce yields by 7 to 54 percent depending on climatic factors and cultivar susceptibility [11]. In addition, pink stem borer (Sesamia inferens (Walker)), and finger millet root aphid (Tetraneura nigriabdominalis (Sasaki)] are notable insect pests of the crop [12, 13].

Spatial variation and GxE interaction are common in varietal selection field trials and are a central problem confronting a plant breeder when comparing the varieties’ genetic potential. These two factors can strongly bias variety estimates and result in large standard errors. Plant breeding trials usually involve a large number of test entries covering extensive areas where spatial variation is likely to confound reliable prediction of genetic values. This is particularly challenging in early generation variety trials with limited genetic material and replications [14]. Efficient approaches that account for more complex environmental variation require complementing experimental designs with appropriate models of analysis [15–17].

Spatial analysis describes an analysis in which the variance of each trial is examined and a suitable structure is used to estimate the trial’s effects. Rather than obviate the need for a good experimental design, this approach increases it, because once a treatment effect is confounded with an environmental effect, the two cannot be disentangled [18]. Factor analytic multiplicative mixed (FAMM) models were used by Smith et al. [19, 20] to extend the genotype and genotype × environment (GGE) analysis. Its significance for the estimate of the related variance structure for GxE effects is a crucial component of the factor analytic (FA) model for multi-environment trials (MET) data, since it offers a good and sparse approximation to the unstructured form and is generally more computationally robust [21].

The use of efficient statistical models to produce precise and illuminating results has advanced MET data analysis, which has a long history with the earlier statistical techniques of analysis of variance (ANOVA) [20, 22–24]. Therefore, it is important to evaluate finger millet genotypes using more efficient statistical methods and mixed model approaches (factor analytic and spatial models under a linear mixed model framework). This study aimed to improve selection strategies in finger millet breeding in Ethiopia through modeling of spatial field trends and the genotype by environment (GxE) interaction.

## Materials and methods

### Study site and experimental design

**Fig 1:**
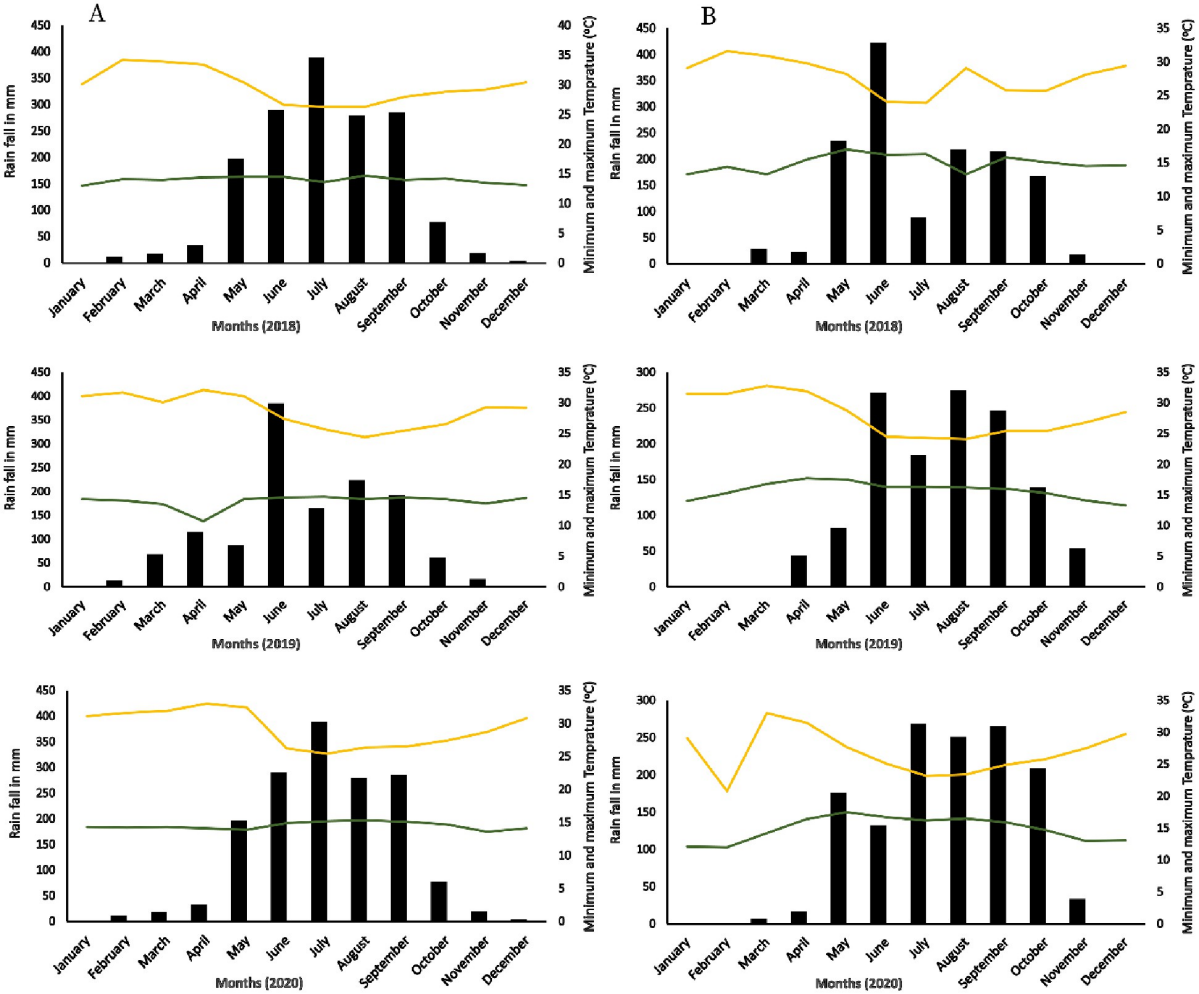
Rainfall, relative humidity, and minimum and maximum temperatures of (A) Bako Agricultural Research Center and (B) Assosa Agricultural Research Center from 2017 to 2020. (Source: Bako and Assosa Agricultural Research Center/Ethiopia).

The genotypes were evaluated for yield at Bako (9º6’ N; 37º9’ E) and Assosa (10°02’ N; 34°34’ E) Agricultural Research Centers in Ethiopia from 2018 to 2021 in seven environments (location—year combinations). The summary of the trials are presented in Table 1, and common genotypes between environments are shown in Table 2. Bako Agricultural Research Center (BARC) receives an annual rainfall of 1,600 mm, with mean maximum and minimum temperatures of 29 ºC and 13 ºC, respectively. The mean monthly relative humidity varies from 46 to 57%, while the main rainy season from May to October, with the most rain received in July and August and short rains also received from March to June. The Assosa Agricultural Research Center (AARC) receives an annual rainfall of 1100 mm, with mean maximum and minimum temperatures of 32 ºC and 17°C, respectively. The soil of the area is Nitosol, which is characteristically reddish to brown in color. The trends of weather data for Bako and Assosa during the study period are summarized in Fig 1. In this study, seven MET datasets were used. To minimize the special variability in determining the genetic value, all trials were conducted using a RCBD with two replications laid out in a rectangular (row x column) array of plots. Trial and environment are terms used synonymously in this study, where environment/trial refers to a year by location combination. Similarly, we interchangeably use to entry, genotype, and variety. The trait we focused on in this study is grain yield, which is expressed in tons per hectare.

**Table 1:** A Summary of trials.

	Row	Range	Genotype	MeanTrait_YLD	Missing
Assosa 2018	7	14	45	0.5	0
Assosa 2019	21	3	21	1.56	0
Assosa 2020	3	13	13	1.43	0
Assosa 2021	13	3	13	1.38	0
Bako 2018	7	14	45	0.94	0
Bako 2020	3	13	13	2.54	0
Bako 2021	13	3	13	0.37	0

**Table 2:** Common genotypes between environments.

	Assosa2018	Assosa2019	Assosa2020	Assosa2021	Bako2018	Bako2020	Bako2021
Assosa2018	45	20	12	11	45	12	11
Assosa2019	20	21	13	12	20	13	12
Assosa2020	12	13	13	12	12	13	12
Assosa2021	11	12	12	13	11	12	13
Bako2018	45	20	12	11	45	12	11
Bako2020	12	13	13	12	12	13	12
Bako2021	11	12	12	13	11	12	13

### Statistical models

A general model for the analysis of GxE data can be built by stacking the data vectors for each trial, $y^{\left(nx1\right)}={\left[y_{1}^{\prime }\ldots y_{n}^{\prime }\right]}^{\prime }$, where $n=\sum_{j=1}^{t}\;n_{j}$, for the ***j***^***th***^ trial. In similar manner, all the fixed and random effects in the model as well as the residuals are combined across trials. In general, consider a series of ‘t’ trials in which ‘m’ varieties are grown (all trials are not necessarily balanced for varieties). A model for *y*^*nx*1^, the vector of individual plot measurements combined across trials, can be written as

$$y=X\tau +Z_{g}u_{g}+Z_{p}u_{p}+e$$

Where $\tau ={\left[\tau_{1}^{\prime }\ldots \tau_{t}^{\prime }\right]}^{\prime }$ is the vector of fixed effects including an overall mean for each of the t trials as well as other trial-specific fixed effects, with associated design matrix, ***X*** = [***X***_**1**_ … ***X***_***t***_], assumed to be full rank. A random effect including a vector of genotype by environment effects, $u_{g}^{\left(mt\;x\;1\right)}={\left[u_{g1}^{\prime }\ldots u_{gt}^{\prime }\right]}^{\prime }$, with associated design matrix $Z_{g}^{\left(nxmt\right)}={\left[Z_{g1}^{\prime }\ldots u_{gt}^{\prime }\right]}^{\prime }$, representing the presence of genotype in each environment. Note that the design matrix may contain columns whose elements are all zero if the associated genotype is not present in that trial. The additional (non-genetic) random effects, ***u***_***p***_ are formed from the extraneous trend effects in each environment (for example, random rows and columns effects) with associated design matrix ***Z***_***p***_, and $e^{\left(nx1\right)}={\left[e_{1}^{\prime }\ldots e_{t}^{\prime }\right]}^{\prime }$ is the vector of residual errors across all trials, and it is assumed that.

$$\left(\begin{array}{c} u_{g}\\ u_{p}\\ e \end{array}\right)\;\sim N\left[\left(\begin{array}{c} 0\\ 0\\ 0 \end{array}\right),\left(\begin{array}{ccc} G_{g} & 0 & 0\\ 0 & G_{p} & 0\\ 0 & 0 & R \end{array}\right)\right]\;,$$

where ***G***_***g***_ is variance matrix for genotype by environment effects, ***G***_***p***_ is the variance matrix of extraneous trend effect and *R* is the variance matrix of the residual errors. In the model, the variance parametric in these variance matrices are directly estimated using REML estimation method. The model can be fitted to unbalanced (an incomplete genotype by environment table) due to the assumption that ***u***_***g***_ is random, which means that interaction effects can be predicted in the absence of data.

### Models for the residual effects: *e*

Models for the variance matrix of the residual effects ***R***, are considered. In the mixed model MET formulation, the vector for residual effects, *e*, can be partitioned into residual effects within individual trials, $e={\left[e_{1}^{\prime }\ldots e_{t}^{\prime }\right]}^{\prime }$, where $e_{j}^{\left(n_{j}\;x\;1\right)}$ is the vector of residual effects for the ***j***^(***th***)^ trial. The subsectors in *e* are assumed to be mutually independent, with variance matrices ***R***_***j***_, where

$$R=\;{⊕}_{j=1}^{t}R_{j}=\left[\begin{array}{cccc} R_{1} & 0 & \ldots & 0\\ 0 & R_{2} & \cdots & 0\\ \vdots & \vdots & \ddots & \vdots \\ 0 & 0 & \cdots & R_{t} \end{array}\right]$$

The model also extends the definition of the R-structure to be a direct sum structure. The residual variance matrix for each trial may be $R_{j}=\sigma_{j}^{2}I_{nj}$ if independence is assumed between field plots.

More frequently, a spatial approach is used using the [25] techniques. Assume that the trial is arranged as a rectangular array of plots in the field as for the row-column design. The variance matrix of the residuals *R*_*j*_ can then be formed as the kronecker product of the variance structure in the row Σ_*r*_ and column E_*c*_ directions.


$$R_{j}={\left(\Sigma_{c}⊗\;\Sigma_{r}\right)}_{j}$$

The most common spatial variance model for the smooth trend in field trials is a separable autoregressive process in the field row and column directions, where Σ_*r*_ and Σ_*c*_ are the *n*_*c*_ *x n*_*c*_ and *n*_*r*_ *x n*_*r*_ correlation matrices in the column and row directions respectively [25]. The variance matrix of the residuals for *j*^*th*^ trial is

$$R_{j}=\sigma_{j}^{2}{\left(\Sigma_{c}\left(\rho_{c}\right)⊗\;\Sigma_{r}\left(\rho_{r}\right)\right)}_{j}$$

### Models for the extraneous trend effects: *u*_*p*_

To model extraneous trend in a MET, the random effects, *u*_*p*_, can be partitioned into sub- vectors $u_{pj}^{\left(b_{j}\;x\;1\right)}$, for each of *j* = 1 ⋯ *t* trials, where *b*_*j*_ is the number of random terms for trial *j*. These random terms are typically based on replicate blocks or rows and columns in the field. In the MET, the sub-vectors in *u*_*p*_ are assumed to be mutually independent, with variance matrix *G*_*pj*_ for trial j, with the block diagonal form given below. That is, there is a variance matrix for the set of extraneous effects for each trial, where

$$G_{p}=\;{⊕}_{j=1}^{t}G_{pj}=\left[\begin{array}{cccc} G_{p_{1}} & 0 & \ldots & 0\\ 0 & G_{p_{2}} & \cdots & 0\\ \vdots & \vdots & \ddots & \vdots \\ 0 & 0 & \cdots & G_{p_{t}} \end{array}\right]$$

The most common form for the variance matrix of these extraneous effects is a simple variance component structure, where $G_{pj}=\sigma_{j}^{2}I_{bj}$.

### Models for the *GxE* effects: *u*_*g*_

The random effects for genotypes, are combined across trials to form the random genotype by environment effects, as $u_{g}={\left[u_{g_{1}}^{\prime }\ldots e_{g_{t}}^{\prime }\right]}^{\prime }$. It is assumed that the associated variance structure has the separable form

$$G_{g}={\text{G}}_{e}⊗{\text{G}}_{v}$$

where *G*_*e*_ and *G*_*v*_ are symmetric *t* × *t* and *m* × *m* scaled variance matrices. That is, *G*_*e*_ holds the (scaled) variances of genetic effects at each environment and covariances of genetic effects between environments, and *G*_*v*_ holds the (scaled) variances of environment effects for each genotype and the covariances of environment effects between genotypes.

An assumption traditionally made in MET analyses of plant breeding trials is that effects between genotypes are independent, so *G*_*v*_ = *I*_*m*_, then *var* (*u*_*g*_) = *G*_*e*_ ⊗ *I*_*m*_. A simplistic model for *G*_*e*_ sometimes used in practice is to consider the results of each trial analysis individually. This is equivalent to a model that allows for heterogeneous genetic variances across environments, but assumes independence between environments.

A more plausible model for the variance matrix of genetic effects is one which includes co- variance to model similarity in genetic performance between environments. In practice, it has been found that most MET data have heterogeneous covariance between environments as well as heterogeneous genetic variances [26, 27].

Based on a factor analytic model, Smith et al. [19] proposed an alternative parsimonious model for variance covariance structure, *Ge*. This model captures the nature of heterogeneous variances and covariances found to occur in most MET data.

### The factor analytic model

In order to model the GxE effects, Smith et al. [19] specified a multiplicative model of factor analytic (FA) form (order k) as

$$u_{g}|f=\left(\Lambda ⊗I_{m}\right)f+\delta$$

where *Λ*^(*t*×*k*)^ is a matrix of environmental loadings for *t* environments, *f*^(*mk*×1)^ is a vector of genotype factor scores for *m* genotypes, and *δ*^(*mt*×1)^ is a vector of residual genotype by environment effects.

It is assumed that

$$\left(\begin{array}{c} f\\ \delta \end{array}\right)\sim N\left(\left[\begin{array}{c} 0\\ 0 \end{array}\right],\left[\begin{array}{cc} I_{k}⊗I_{m} & 0\\ 0 & \Psi ⊗I_{m} \end{array}\right]\right)$$

where *Ψ*^(*t*×*t*)^ is a matrix of specific variances, modelling the residual *GxE* variance not explained by the factor model.

The variance of *u*_*g*_ is again of the form ***G***_***g***_ = ***G***_***e***_ ⊗ ***I***_***m***_, where

$$G_{e}=\Lambda \Lambda^{\prime }+\Psi$$

The form of the variance matrix of genetic effects, ***G***_***e***_, in a factor analytic model is comprised of two parts. Products of the factor loadings ***ΛΛ***′ give the portion of the genetic variance explained by the factor or regression model. The inclusion of the term, ***δ*** for residual ***GxE*** captures lack of fit to the factor model, and ***Ψ*** is the matrix of specific variances not accounted for by the loadings in the FA model. This FA model accommodates both heterogeneity of genetic variance across environments (due to ***Λ*** and ***Ψ***) and heterogeneity of genetic covariance, or correlation, between pairs of environments (due to ***Λ***).

### Heritability formula

The heritability (H^2^_j_) value for the j^th^ trial was determined using an extended technique that takes unbalanced data into account, as suggested by Cullis et al, [28].

$$H_{j}^{2}=1-\frac{A_{j}}{2\sigma_{gj}^{2}}$$

Where Aj is the average pairwise prediction error variance of genotype effects for the j^th^ environment, and σ^2^_gj_ is the genetic variance at environment j.

## Results

### Spatial analysis

Individual trials were subjected to separate analyses to account for non-genetic effects using the Gilmour et al. approach (1997) [25]. The initial model fitted for each trial was a randomized complete block (RCB) model with random block/replication and variety effects, and the residual correlation structure is denoted by id(Column):id(Row), where id refers to the identity matrix. This model reflects the RCB analysis based on a mixed model approach. The three key types of spatial variation were then considered over the RCBD model. Thus, a spatial model for the residuals using a separable autoregressive process in the column and row dimensions was first fitted over the RCB model for the local variation. After that, the spatial model for extraneous variation was fitted along the column and row while keeping significance terms for local variation. The Residual Maximum Likelihood Ratio (REMLR) test was used to determine whether each fitted spatial model was significant for both local and extraneous variation. Finally, the Wald test was used to test the spatial models for global variation.

**Fig 2:**
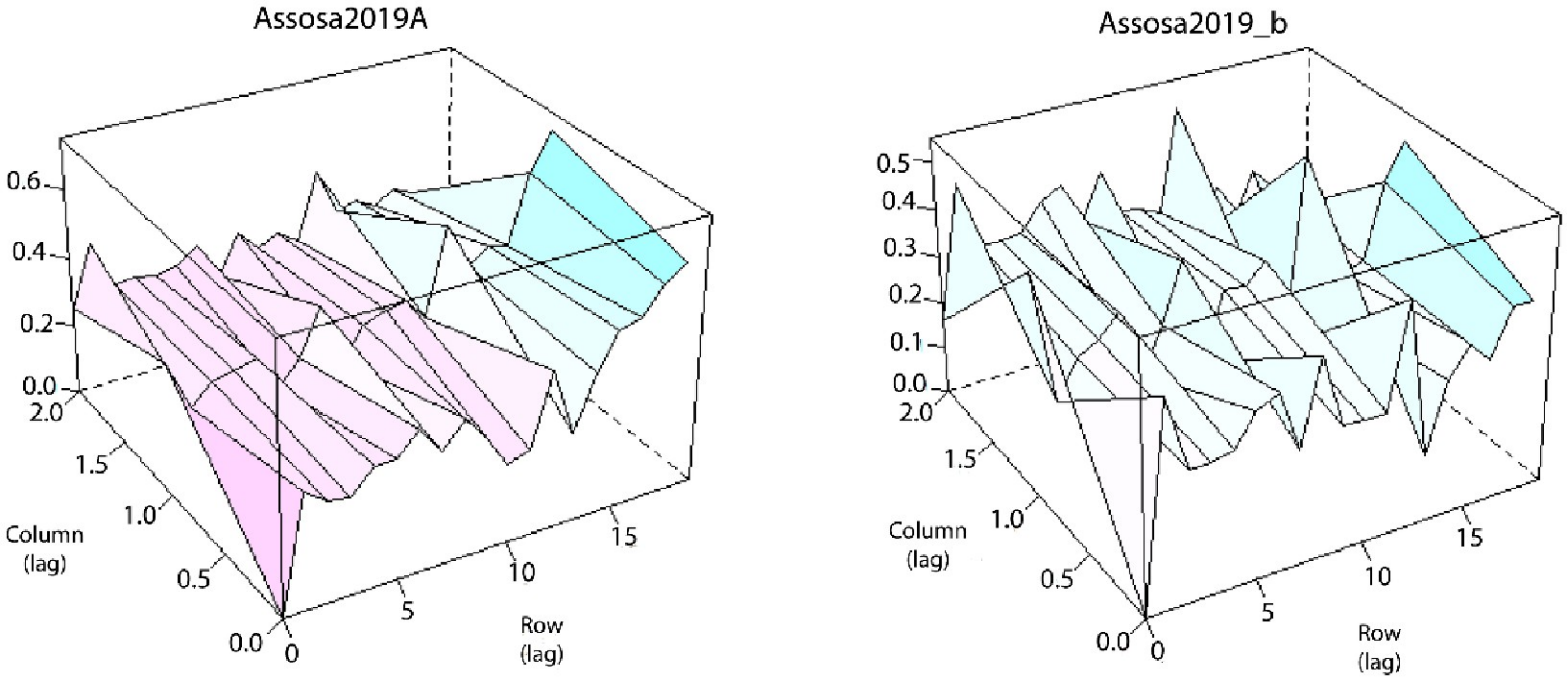
Sample variogram plot of the residuals at trial Assosa2019 from RCBD model (Assosa2019_a) and from global spatial model (Assosa19_b).

The presence of global and extraneous variation associated with the row and column direction can be checked with the help of a model diagnosis tool, in particular the sample variogram [25], which was used as a typical model diagnostic plot for trial Assosa19 using the Metplot package, which is a part of ASReml-R, and is depicted in Fig 2. In the row direction, the sample variogram plot (Assosa19_a) showed evidence of a global trend, with the variogram steadily increasing. This trend, as demonstrated by Stefanova et al. [29], is most likely the presence of soil fertility or other spatial gradients of the trial, implying confounded fixed covariate effects. This global trend may be accounted for in the model by employing linear or polynomial regression over a centered row number, denoted lrow. After taking into account the overall trend in the row direction, the sample variogram (Assosa19_b) in Fig 2 indicated a drop from a steadily increasing trend in the row direction.

Table 3 presents the Wald test for global variation and the REMLR test for local and extraneous spatial variation. The spatial variations were appropriately modeled for the trials Assosa2018, Assosa2019, Assosa2021, and Bako2018. Thus, the local spatial trend was found to be significant for Assosa2018 and Assosa2021 along the row direction (p-value = 0.007 and 0.019, respectively), and for Assosa2021 along the column direction (p-value = 0.033). The global spatial variation was found to be significant along the column direction at Assosa2018 and Bako2018 (p-value = 0.006 and 0.011, respectively). This demonstrated how spatial variation is a crucial component of plant breeding trials that should be considered during variety trails at multi locations. The estimates of the local spatial correlation parameters at trial Assosa2021 and Bako2018 were positive (ρ2 = 0.52 and ρ3 = 0.26, see Table 3), suggesting that plots that were close together were more likely to be comparable than plots that were further apart. The negative spatial correlation value at trial Assosa2018 (ρ2 = -0.57) implied inter-plot competition, as demonstrated by Stringer and Cullisin [30].

**Table 3:** Summary of spatial analysis: Spatial variation, fitted model term, Wald and REMLR test statistic and P-value.

Trial name	Spatial variation	Model terms	Wald^a^ /REMLR^b^ test statistic	P-value
Assosa2018	Local	id(Column):ar1(Row)	7.29	0.007
	Global	lcol	9.64	0.006
Assosa2019	Global	lrow	4.22	0.045
Assosa2020	-	-	-	-
Assosa2021	Local	id(Column):ar1(Row)	5.49	0.019
Bako2018	Local	ar1(Column):id(Row)	4.56	0.033
	Global	lcol	7.87	0.011
Bako2020	_	-	-	-
Bako2021	-	-	-	-
	ρ1 = -0.57, ρ2 = 0.52, ρ3 = 0.26			

^a^Test for global trend after significant terms for extraneous variation and local trend were fitted

^b^Test for extraneous variation and local trend after significant terms for global trend were fitted

ρ1, ρ2 and ρ3 are estimates for autoregressive order 1(AR1) spatial correlation parameters at Assosa2018, Assosa2021 and Bako2018, respectively, and trials with empty cell (-) indicated that no spatial variation was observed.

### Factor analysis

The FA models were considered for genotype by environment (GxE) analysis while keeping the spatial models provided in the individual trial analysis. The adequacy of the FA models of several k orders was formally assessed as it was fitted within a mixed model framework based on the percentage of GxE variance explained by the factor components. Table 4 presents the results from the factor analysis. It includes the total percentage of (GxE) variance explained by the model’s factor components for each trial as well as for all trials. Except for trial Bako2021, the FA models fit almost all trials well, and the genetic variance was well explained by the two factor components. Overall, the two multiplicative terms of the factor analytic models accounted for nearly 98 percent of the GxE variance, with the first multiplicative term accounting for 93 percent. Bako2021’s insufficient fit with the FA model suggests that the trial is not as well correlated as some of the other trials [31].

**Table 4:** Results from fitting FA model.

Trials	Factor1	Factor2	All
Assosa2018	91.62	1.52	93.15
Assosa2019	100	0	100
Assosa2020	86.1	13.9	100
Assosa2021	83.5	16.5	100
Bako2018	10.01	89.99	100
Bako2020	96.61	0.61	97.22
Bako2021	6.94	16.19	23.13
%var FA-1 = 93.12,		% var FA-2 = 97.97	

%varFA-1 = percentage of GxE variance explained by fitting FA-1; %varFA-1 = percentage of GxE variance explained from fitting FA-2 model

**Fig 3:**
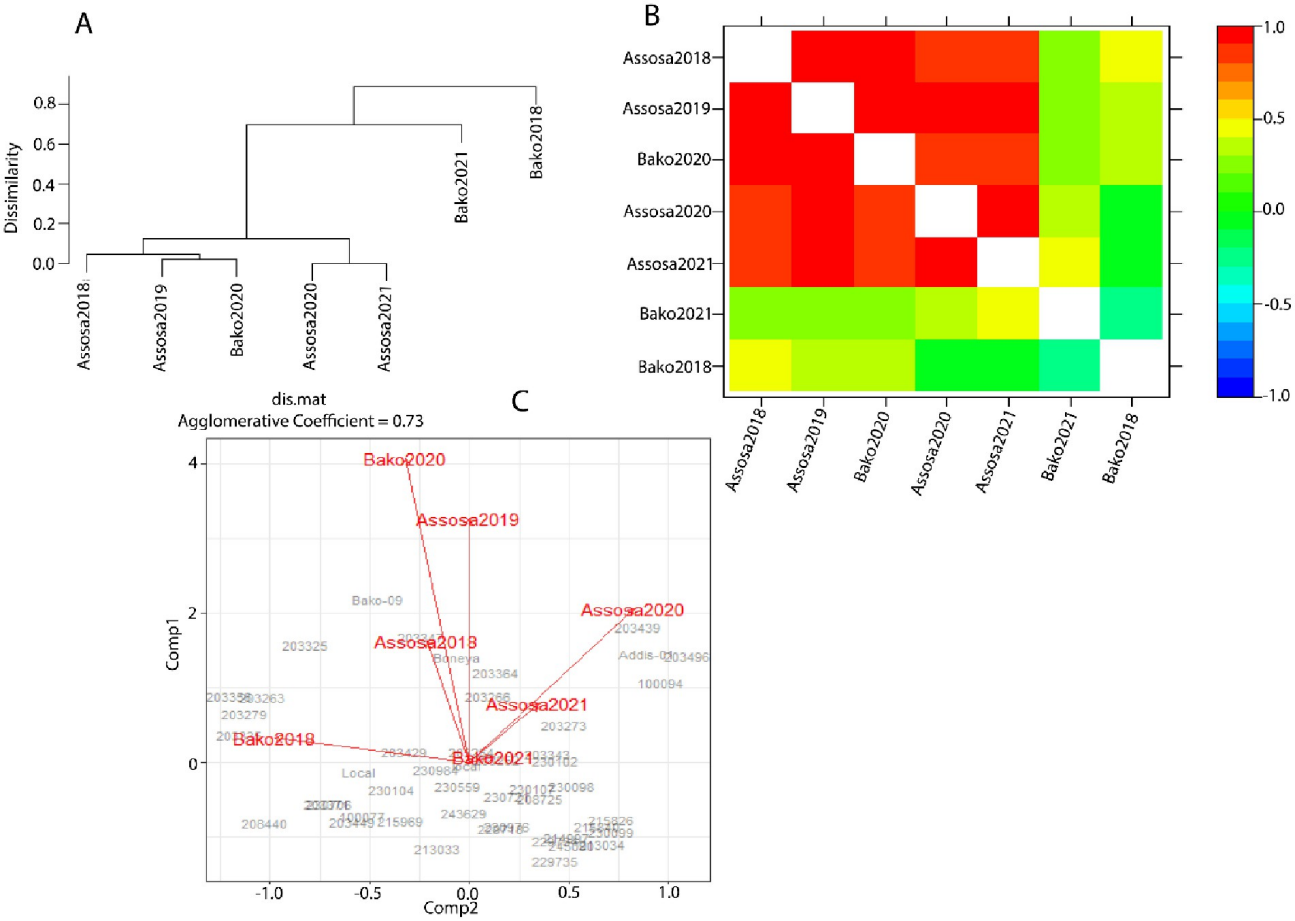
Dendrogram of the dissimilarity matrix (a), heat map representation of the genetic correlation matrix (b) and the bi-plot(c).

The other important result from factor analysis was a cluster analysis using a dendogram, shown in Fig 3(a), which grouped trials based on genetic correlation between them. Based on the Cullis et al. [31] suggestion that clusters formed at the dissimilarity cut-off (approximately below 0.6), the dendrogram suggested possibly three clusters of trials, where the first cluster was comprised of at most five trials. This showed that the genotype ranking was almost similar for all trials found within these formed clusters, and a different ranking of genotypes for the trials found in different clusters. Genotype selection, therefore, was performed for each cluster using average BLUPs as a selection index, provided that the formed clusters were reasonably justified for making genotype selection independently for each of the clusters.

In addition to the dendrogram, other typical summaries from the factor analysis included a heatmap of the genetic correlations between all trials. This is presented in Fig 3(b), which shows the different correlation patterns between trials. From the heatmap, we can see most of the trials were highly correlated. This indicated that genotype selection can be performed by averaging genotype means across nearly all trials in the first cluster indicated in red. There were also some trials that showed negative genetic correlations, such as Assosa2018 and Assosa2021(Table 5), suggesting that there may have been a reversal action in genotype rankings among these negatively correlated trials. The bi-plot in Fig 3(c) also demonstrated the concept of variety performance and stability across environments, as well as the discriminating power of a trial. Trials with a long arm from the center of the bi-plot had a high genetic variance compared to the others, and thus had a relatively high discriminating power for genotypes. Thus, Bako2020 and Assosa2019 had high genetic variance compared to the others. Therefore, based on the dendrogram, heat-map and bi-plot (Fig 3) as well as the genetic correlation from Table 5, we considered 3 clusters of trials (C1, C2 and C3) for trait yield, where Assosa2018, Assosa2019, Assosa2020, Assosa2021, and Bako2020 were in C1; Bako202 in C2; and Bako2018 in C3. An average of BLUPs was used as selection index to choose superior and stable varieties through ranking average BLUPs within clusters.

**Table 5:** Genetic correlation between environments.

	Assosa	Assosa	Assosa	Assosa	Bako	Bako	Bako
	2018	2019	2020	2021	2018	2020	2021
Assosa2018	1						
Assosa2019	0.957	1					
Assosa2020	0.842	0.928	1				
Assosa2021	0.825	0.913	0.999	1			
Bako2018	0.42	0.317	-0.06	-0.096	1		
Bako2020	0.95	0.983	0.883	0.866	0.385	1	
Bako2021	0.202	0.263	0.394	0.404	-0.298	0.227	1

### Variance components

The genetic variance, error variance, and heritability for each trial from the final fitted Spatial+FA models are presented in Table 6. The estimates for variance component parameters ranged from 0.003 to 0.477 for genetic variance, from 0.012 to 0.362 for error variance and from 34.32 to 95.24 for heritability. Two of the seven trials, Bako2020 and Assosa2019, had higher genetic variance for yield. This indicated that these testing locations had relatively high discriminating power for genotypes. This may have been attributed to substantially higher amounts and distribution of rain for Assosa and Bako in 2019 and 2020, respectively. This also emphasized the significance of taking meteorological data from a specific cropping season into account when recommending the best genotype for a given cropping season, as well as its broader application across the country’s many agro-ecologies. In addition, the Bako2021 and Bako2018 trials were found to be poor trials with low genetic variance and heritability, which could also be attributed to low rain fall or drought at Bako in 2021. This also led us to exclude the BLUPs of these trials when averaging across trials for selecting superior genotypes.

**Table 6:** Variance component results MET analysis using spatial and FA models.

	GeneticVariance	ErrorVariance	met.Hsq
Assosa2018	0.081	0.012	95.24
Assosa2019	0.295	0.362	92.9
Assosa2020	0.137	0.089	91.94
Assosa2021	0.021	0.035	89.91
Bako2018	0.03	0.149	46.41
Bako2020	0.477	0.314	93
Bako2021	0.003	0.026	34.32

**Fig 4:**
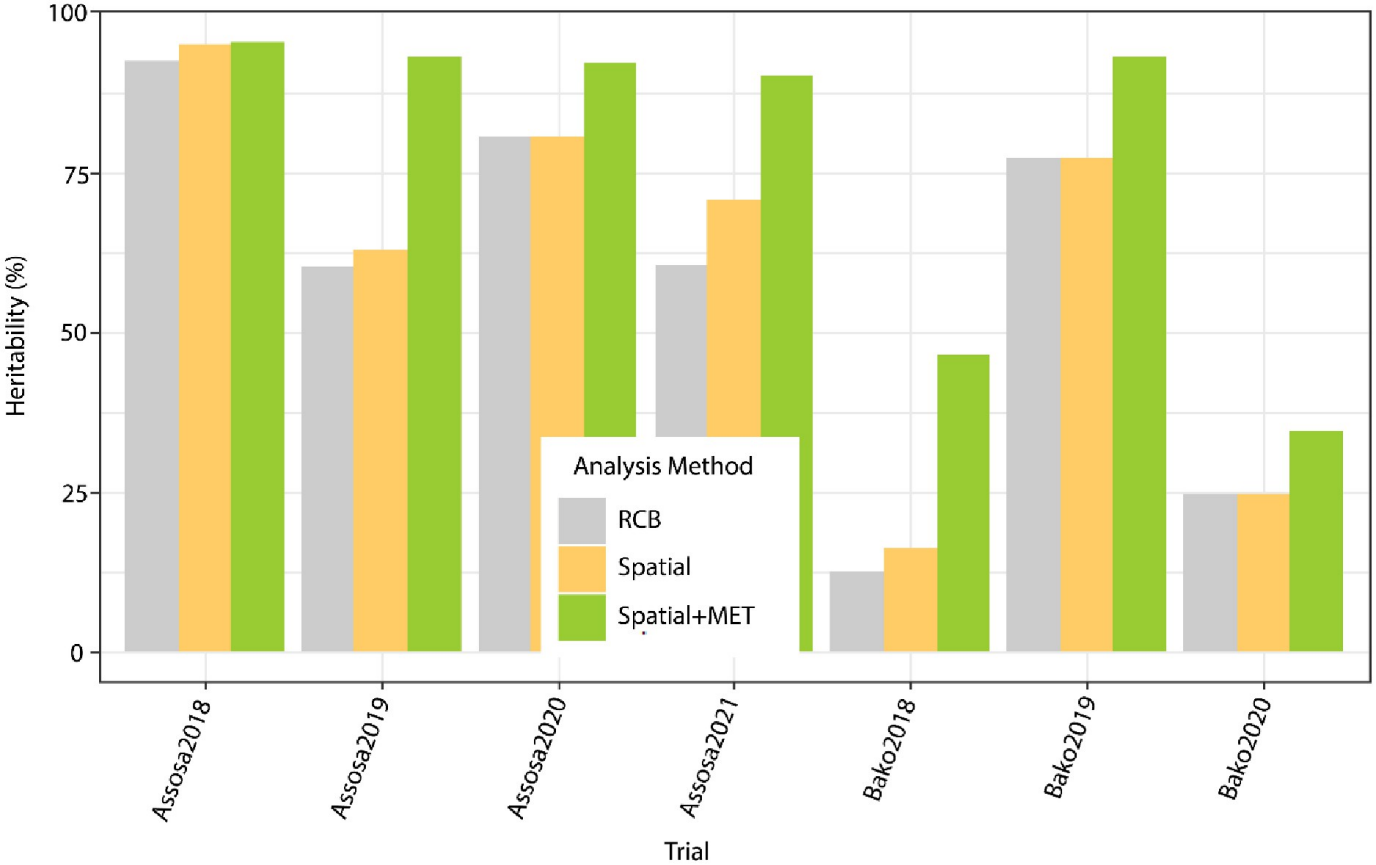
Improvements in heritability through the applications of spatial and FA models.

The heritability of yield at each trial is shown in Fig 4 using conversional, spatial, and spatial+FA analysis. It shows that using spatial analysis improved heritability, and that using spatial+FA analysis improved heritability even more. In general, analyzing MET data with spatial and FA models improved genotype evolution precision and accuracy by capturing non-genetic variation associated with agricultural field experiments and appropriately exploiting the information stored in the MET dataset [31, 32].

### BLUPs for genotypes mean values across environments

A standard method for estimating random effects in a mixed model is best linear unbiased prediction (BLUP), which has the property of minimum mean square error of prediction, and they can form a more accurate estimation of the underlying effects. Genotype effects are mostly fitted as random terms in a plant breeding context where accuracy of genotype ranking is important for selection of superior genotypes. This is more required in the early stages of genotype trials conducted with a large number of entries. The performance of genotypes can be ranked based on the values of BLUPs averaged across correlated environments of the 1st cluster (C1), excluding Bako2021 and Assosa2019 since they showed low genetic correlation with the other trials and low genetic variance. Table 7 shows that more than 28% (13) of the 46 genotypes had average grain yields greater than 1.3 t/ha. However, the predicted mean grain yield indicated three genotypes with a higher mean yield than 1.6 t/ha: one was the check, Bako-09, and the other two were 203439 and 203325 (Table 7). BLUP analysis further indicated that Bako trials in 2020 and Assosa trials in 2019 yielded high grain yields, implying that these sites are the best testing locations for distinguishing between finger millet genotypes and the most suited agro-ecologies for finger millet crop production in general.

**Table 7:** BLUPs for genotype means across cluster 1 (C1) of correlated environments.

Genotype	Bako	Bako	Assosa	Assosa	Assosa	Assosa	Bako	Average for
	2018	2020	2018	2019	2020	2021	2021	C1
Bako-09	1.141	3.419	1.094	2.435	1.813	1.523	0.361	1.867
203439	0.908	3.059	0.938	2.234	1.867	1.55	0.445	1.693
203325	1.166	2.978	1.025	2.106	1.556	1.422	0.368	1.681
203347	1.078	2.994	0.955	2.164	1.673	1.47	0.404	1.675
Boneya	1.033	2.829	0.901	2.018	1.606	1.445	0.384	1.592
Addis-01	0.881	2.804	0.885	2.038	1.749	1.506	0.404	1.580
203496	0.845	2.764	0.843	2.023	1.768	1.514	0.401	1.555
203364	0.991	2.648	0.829	1.902	1.558	1.428	0.353	1.507
203356	1.192	2.581	0.779	1.736	1.269	1.31	0.336	1.455
203263	1.164	2.493	0.833	1.728	1.287	1.318	0.371	1.444
100094	0.848	2.558	0.722	1.834	1.631	1.461	0.39	1.440
203266	0.98	2.466	0.782	1.733	1.447	1.386	0.38	1.419
203279	1.167	2.372	0.696	1.603	1.196	1.283	0.358	1.358
203273	0.897	2.186	0.621	1.526	1.369	1.358	0.382	1.268
203335	1.155	2.184	0.625	1.455	1.099	1.245	0.346	1.266
203429	1.008	1.984	0.579	1.329	1.134	1.263	0.36	1.176
203264	0.952	1.903	0.542	1.329	1.181	1.283	0.394	1.145
203343	0.889	1.923	0.507	1.311	1.222	1.301	0.375	1.135
203252	0.926	1.884	0.487	1.269	1.161	1.276	0.369	1.115
230102	0.878	1.866	0.483	1.268	1.2	1.293	0.375	1.107
local	0.989	1.854	0.428	1.206	1.062	1.236	0.358	1.083
230984	0.968	1.812	0.462	1.199	1.074	1.241	0.361	1.079
230104	0.991	1.646	0.425	1.057	0.953	1.194	0.352	1.004
230559	0.939	1.658	0.395	1.08	1.014	1.219	0.36	1.002
230098	0.845	1.623	0.377	1.077	1.091	1.252	0.372	0.984
230107	0.876	1.617	0.375	1.063	1.055	1.237	0.367	0.981
230721	0.891	1.546	0.346	1.001	0.998	1.214	0.363	0.945
203371	1.034	1.532	0.37	0.951	0.841	1.149	0.343	0.944
230706	1.031	1.526	0.351	0.947	0.84	1.149	0.343	0.937
208725	0.862	1.523	0.335	0.99	1.015	1.222	0.366	0.934
100077	0.995	1.405	0.294	0.857	0.807	1.137	0.344	0.875
243629	0.914	1.406	0.286	0.88	0.892	1.172	0.355	0.875
208440	1.07	1.373	0.276	0.81	0.71	1.097	0.332	0.857
203449	1	1.349	0.296	0.822	0.778	1.126	0.33	0.856
215969	0.961	1.356	0.266	0.826	0.814	1.141	0.347	0.849
215826	0.789	1.31	0.241	0.834	0.965	1.204	0.37	0.826
230976	0.869	1.28	0.231	0.788	0.864	1.163	0.358	0.811
229718	0.873	1.254	0.22	0.766	0.845	1.156	0.356	0.798
215840	0.796	1.248	0.215	0.782	0.922	1.188	0.367	0.795
230099	0.78	1.202	0.195	0.748	0.911	1.184	0.368	0.772
214997	0.813	1.162	0.178	0.706	0.853	1.16	0.362	0.752
229734	0.82	1.135	0.167	0.683	0.831	1.152	0.36	0.738
213033	0.91	1.094	0.152	0.624	0.712	1.103	0.346	0.717
213034	0.779	1.09	0.146	0.656	0.846	1.159	0.364	0.715
245090	0.803	1.088	0.147	0.649	0.82	1.148	0.361	0.715
229735	0.805	0.951	0.087	0.535	0.737	1.116	0.356	0.645

## Discussion

Following advances in statistical science and knowledge, methods of data analysis for crop variety evaluation programs have improved over recent years [22, 33, 34]. However, these improved statistical techniques have not yet been well utilized in many crop variety evaluation programs, including finger millet breeding in Ethiopia. This study’s data set was incomplete; that is, not all varieties were grown in all environments. This was problematic for statistical analyses using ANOVA-based approaches, such as AMMI and GGE bi-plot analysis. To compensate for our incomplete data set, we opted to use the linear mixed model framework.

Control by blocking, which is based on observable factors like soil type and topography, is commonly used in on-station trials, but several possible inputs are unknown or unmeasured, which stresses the importance of incorporating spatial information into the analysis of experiments to improve the precision of genotypes evaluation trials. This is especially important for field trials in Ethiopia, where the problem varies from plot to plot, as some findings justify [35]. The spatial mixed model approach proposed by Gilmour et al. (1997) [25] has received particular attention as it simultaneously considers three types of spatial variation to be modeled: local, global, and extraneous variations. Despite the recommendations by several authors, these approaches are not widely used in crop variety evaluation programs as a routine data analysis platform. In this particular study, we took advantage of these approaches and results of the spatial analysis were compared to that of randomized complete block (RCB) analysis. The investigated spatial mixed models showed better data fitting, resulting in a substantial improvement in the estimates of genetic parameters. Furthermore, our spatial analysis detected the presence of global, extraneous, and local trends. These trends could be explained by the presence of diseases and insects within and between blocks, missing plots, improper irrigation distribution, and micro soil fertility spots as well as the experiment’s spatial gradients or the presence of soil fertility, as suggested by Stefanova et al. [29]. It could also be linked to the residual dependence of plots. When spatial trends were taken into account, relevant research on maize [36], sorghum [37], barely [38], potato [39], and soybean [40] likewise produced excellent results.

The ANOVA-based approach always assumes constant variance across environments and no correlation between environments. We conducted MET analysis by fitting heterogeneous variance for residuals and a factor analytic model to the GxE effects, which results in the Restricted Maximum Likelihood (REML) estimates for genetic correlation between environments, and hence cluster analysis using a dendrogram and bi-plot. The results of MET data analysis showed that modeling GE interactions with FA models in combination with models for spatial variations resulted in a significant improvement in the estimates of genetic parameters, and this was demonstrated with evidence of heritability. Results from a related study on sorghum [41], durum wheat [42], and maize [36] revealed that modeling field spatial correlation plus MET through FA combined under a linear mixed model also indicated significant improvement in heritability analysis. The FA models were found to be useful not only for estimating and predicting GxE interaction effects, but also for estimating GxE variance and performing bi-plot analysis. Correlated environments could be identified using the estimated GxE variance, and breeders can select genotypes using BLUPs averaged across correlated environments. The superior varieties were chosen based on the average BLUPs because it could accurately predict the exact ranking of each variety’s performance [32]. Anuradha et al. [43] employed BLUPs analysis via simultaneous selection index. However, their BLUP analysis differed from ours because the BLUP in our case was enhanced by capturing spatial variations and modeling the genetic correlation structure between environments.

We found two clusters of correlated environments using the dendrogram and the bi-plot, which aided selection of superior finger millet varieties within each cluster. Only one cluster was chosen for the entire variety selection because it formed with relatively high heritability and encompassed more environments. The second cluster, on the other hand, was found to have low heritability and fewer environments.

## Conclusion

Combining MET with spatial and FA models allowed for a better understanding of the genetic effect and enhanced the precision and accuracy of genotype evolution by allowing the breeder to also take the GxE interaction effect into consideration. This makes it possible to isolate the genetic effect or further explore the GxE interaction effect, depending on the goal. The genotypes with the highest potential for a further verification study that might be used as a released variety could be found using the fitted information.

## Supporting information

S1 Table Grain yield data. (TXT)
